# Supplementary material for: Biomolecular condensates formed by designer minimalistic peptides
Source: Nat Commun. 2023 Jan 26;14:421. doi: 10.1038/s41467-023-36060-8 (PMC9879991; doi:10.1038/s41467-023-36060-8)
Supplement: Supplementary file 1 — Supplementary Information [file 41467_2023_36060_MOESM1_ESM.pdf]

Supplementary Information

Biomolecular Condensates Formed by Designer Minimalistic Peptides

Avigail Baruch Leshem[1], Sian Sloan-Dennison[2], Tlalit Massarano[1], Shavit Ben-David[1], Duncan Graham[2], Karen Faulds[2], Hugo E. Gottlieb[3], Jordan H. Chill*[3] and Ayala Lampel*[1,4-6]

[1]Shmunis School of Biomedicine and Cancer Research, George S. Wise Faculty of Life Sciences, Tel Aviv University, Tel Aviv 69978, Israel.

[2]Department of Pure and Applied Chemistry, Technology and Innovation Centre, University of Strathclyde, 99 George Street, Glasgow G1 1RD, U.K

[3]Department of Chemistry, Faculty of Exact Sciences, Bar Ilan University, Ramat Gan, 52900, Israel

[4]Center for Nanoscience and Nanotechnology Tel Aviv University, Tel Aviv 69978, Israel.

[5]Sagol Center for Regenerative Biotechnology Tel Aviv University, Tel Aviv 69978, Israel

[6]Center for the Physics and Chemistry of Living Systems Tel Aviv University, Tel Aviv 69978, Israel, Tel Aviv 69978, Israel.

*co-corresponding authors

email: Jordan.Chill@biu.ac.il; ayalalampel@tauex.tau.ac.il

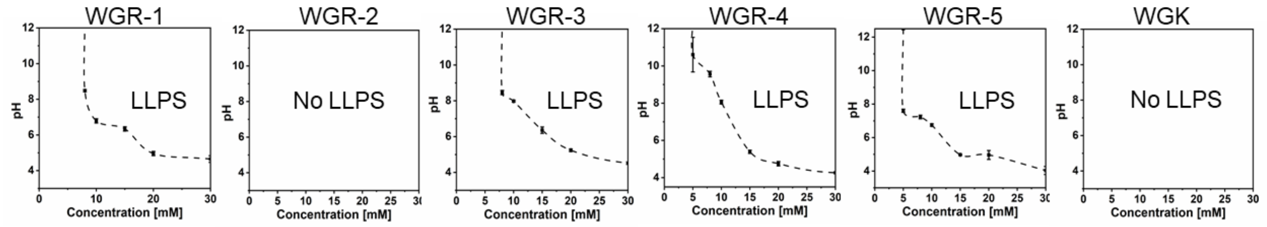

Supplementary Figure 1. Phase diagram of the peptides as a function of peptide concentration and pH, in different buffers: citrate buffer for pH 3-7, tris buffer for pH 7-9, and ammonium bicarbonate buffer for pH 9-12. 0.2 M NaCl was added to all samples, measurements were obtained at room temperature. LLPS was not observed for WGR-2 and WGK. Values represent average pH of n=3 independent measurements, data are presented as mean values +/- SD. Source data are provided as a Source Data file.

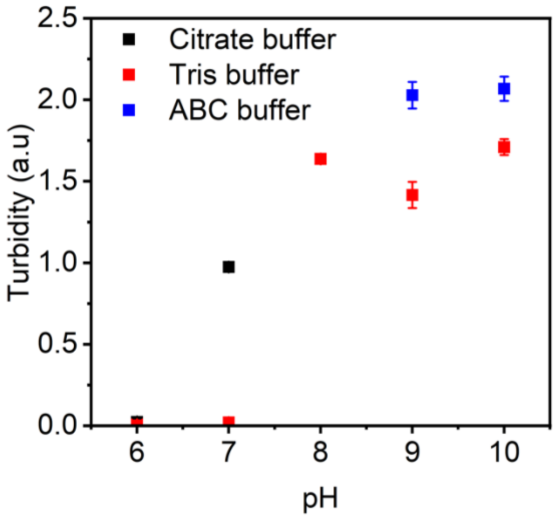

Supplementary Figure 2. Turbidity assay of WGR-1 (10 mM) in citrate buffer (pH 6-7), Tris buffer (pH 6-10) and ammonium bicarbonate buffer (ABC, pH 9-10). Turbidity was monitored at λ=500 nm. Data are presented as mean values of n=3 +/- SD. Source data are provided as a Source Data file.

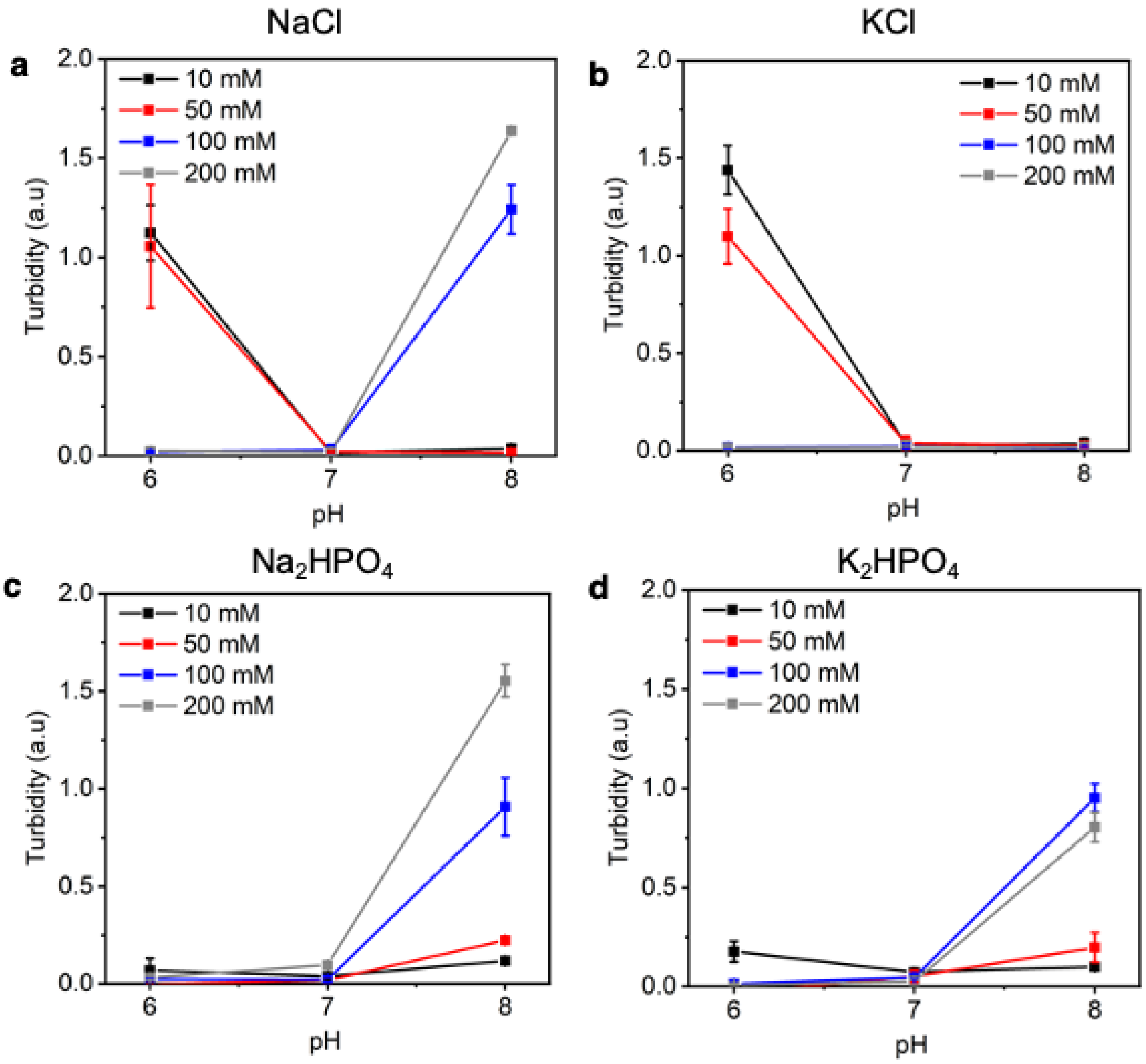

Supplementary Figure 3. Effect of Hofmeister series salts on peptide LLPS. Turbidity assay (λ=500 nm) of WGR-1 (10 mM) as a function of pH with varying concentrations of either NaCl, KCl, Na_2HPO_4 or K_2HPO_4. Data are presented as mean values of n=3 +/- SD. Source data are provided as a Source Data file.

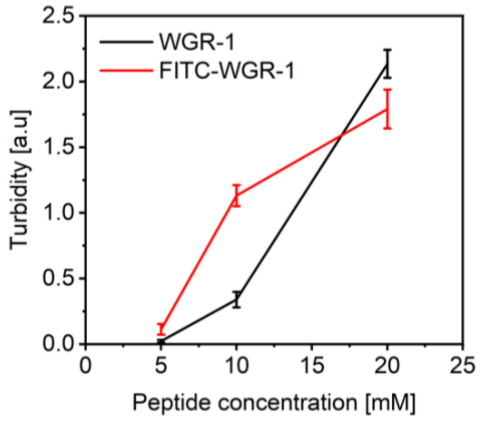

Supplementary Figure 4. Turbidity analysis of varying concentrations of WGR-1 in Tris buffer at pH 8 in the presence of 0.2 M NaCl. Red line indicates 0.5% of FITC-labeled peptide. Data are presented as mean values of n=3 +/- SD. Source data are provided as a Source Data file.

Supplementary Table 1.

**Table 1. Apparent diffusion coefficients in the condensed and dilute phase**				
	**WGR-1**	**WGR-3**	**WGR-4**	**WGR-5**
D condensed phase (FRAP)	4.58E-14	5.53E-14	2.53E-14	1.41E-14
Error	6.05E-15	2.88E-15	7.02E-15	4.71E-15
D dilute phase (NMR)	1.91E-10	2.18E-10	2.23E-10	2.44E-10
Error	0.11E-11	0.12E-11	0.12E-11	0.13E-11

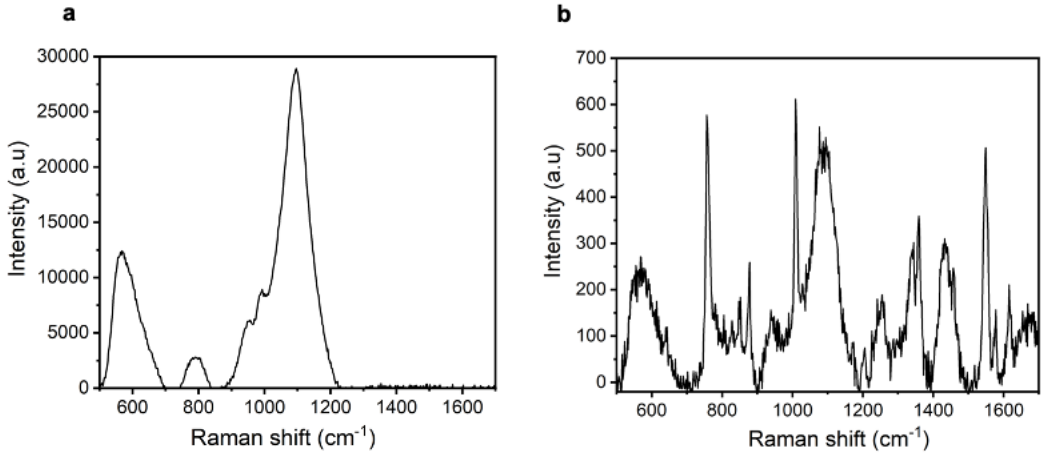

Supplementary Figure 5. a. Raman spectra of peptide droplet buffer control (background). **b**. Raman spectra of dried peptide droplets. Source data are provided as a Source Data file.

a

Notation	Sequence
WGR-6	GRGRGRGWPGVGY
WGR-7	WGRGRGRGPGVGY
WGR-8	WGRGRGRGAPGVGY
WGR-9	GRGRGRGAPGVGY

b

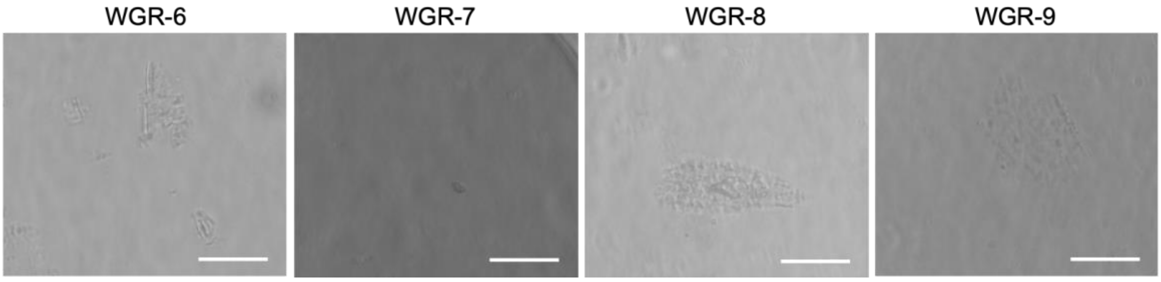

Supplementary Figure 6. a. Table of the 4 sequence variants designed to study the role of Trp in LLPS. **b.** Optical microscopy images of the peptides at 30 mM pH 11 with 0.2 M NaCl. No droplets were observed for all 4 peptides. Scale bars=50 μm.

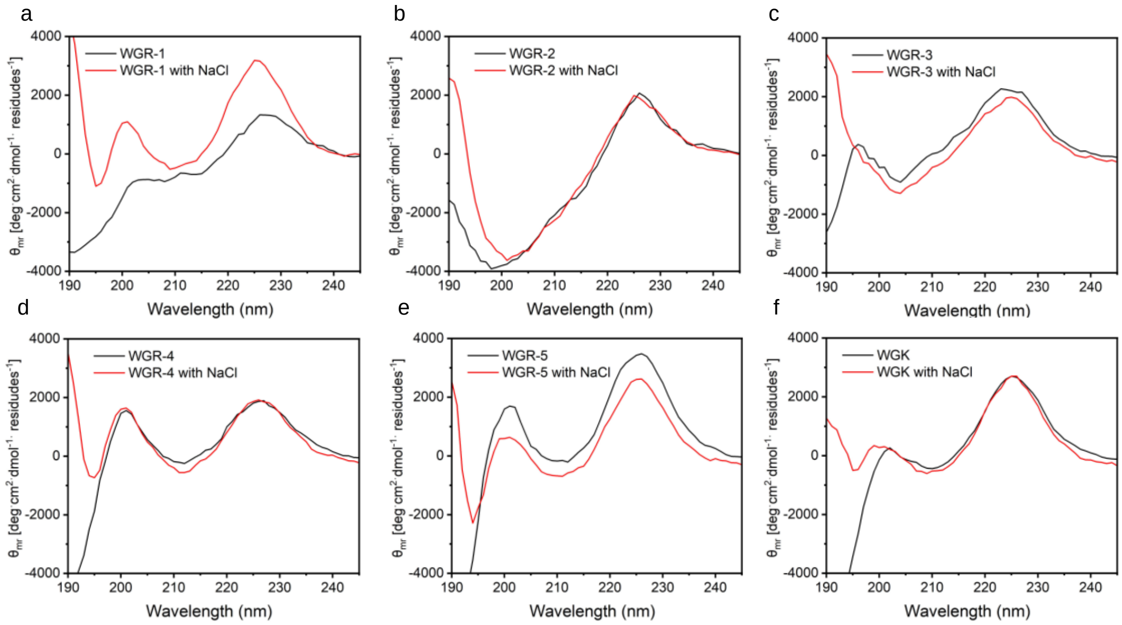

Supplementary Figure 7. CD analysis of peptides at 1 mM with and without NaCl.

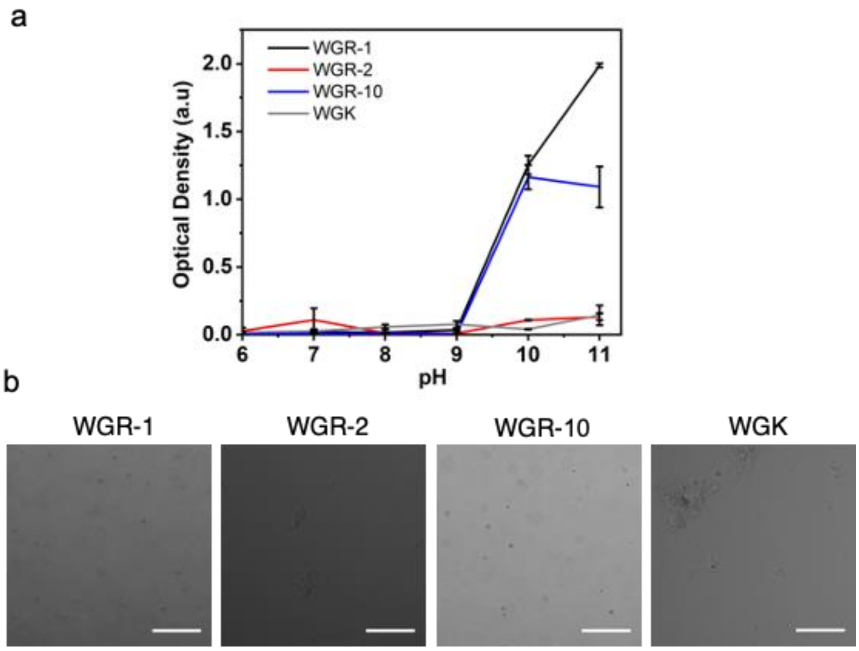

Supplementary Figure 8. a. Turbidity analysis of peptide LLPS at a concentration of 20 mM at varying pH values. WGR-10 contains a Pro/Gln substitution. Each point represents an average of n=3, data are presented as mean values +/- SD **b**. Optical microscopy images of peptides at 20 mM at pH 10. Scale bar=50 μm. Source data are provided as a Source Data file.

Supplementary Information - NMR analyses

Supplementary Table 2. pKa values of LLPS-promoting peptides.

Supplementary Table 2. pKa values of LLPS-promoting peptides.

**Peptide**	**pKa**
WGR-1	7.50 ± 0.01
WGR-3	7.49 ± 0.01
WGR-4	7.37 ± 0.01
WGR-5	7.51 ± 0.01

pKa values were determined by following the chemical shifts of Trp^1-Hα and Trp^1-Hβ at pH values of 6.0, 7.0, 7.5, 8.0 and 9.0. The 10 obtained chemical shifts were fitted to the Henderson-Hasselbach equation with the assumption that peak position is a weighted average of the two end-point chemical shifts; optimization parameters were the two end-point shifts (representing fully ionized and fully deionized forms of the N-terminal amino group of the peptide. Errors were determined by Monte Carlo simulations with a measurement error of 0.01 ppm for all chemical shifts. Data are presented as mean values +/- SD

Supplementary Table 3. Urea analysis.

Supplementary Table 3. Urea analysis.

**Peptide**	**Trp^1-Cα**	**Trp^9-Cα**	**Pro^{10}-Cα**	**Val^{12}-Cα (maj.)**	**Val^{12}-Cα (min.)**	**Tyr/Phe14-Cα**
WGR-1	0.12	0.15	0.05	0.02	0.08	0.37
WGR-2	0.13	0.20	ND[a]	0.09	0.15	NA[b]
WGR-3	0.12	**0.56**	0.06	0.03	0.06	0.33
WGR-5	0.13	0.07	0.04	0.02	0.07	0.36

Values represent chemical shift changes (^{13}C, ppm, on a 700 MHz spectrometer) for representative Cα nuclei along the peptide sequence upon addition of 8 M urea to the sample. All measurements were conducted at 3 mM peptide concentration and maintaining a constant sample volume and temperature (300 K). Values reflecting the intramolecular effect are underlined; the large effect of Phe14 upon Trp9 is in bold.

[a]peak overlaps with the tris buffer peak

[b]Residue 14 is absent in WGR-2

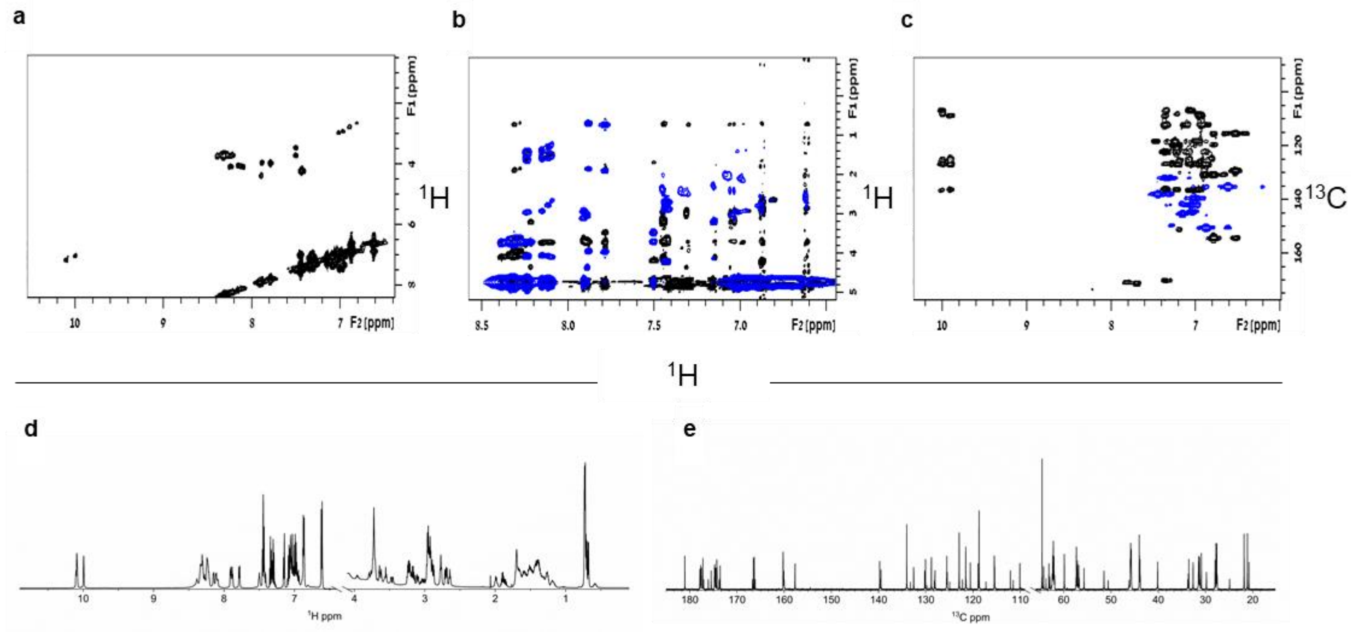

Supplementary Figure 9. 20 mM WGR-1 NMR data at pH 6, 285 K. Shown are spectra used for assignment of all residues of the peptide. **a.** 2D-COSY spectra in the H^{aro}-H^{ali} and H^{N}-H^{ali} region. **b.** Overlay of homonuclear 2D-TOCSY (black) and ROESY (blue) spectra in the H^{aro}-H^{ali} and H^{N}-H^{ali} region. **c.** Overlay of heteronuclear 2D-HMBC (black) and HMQC (blue) spectra in the aromatic region. **d**. The one-dimensional ^{1}H spectrum. **e.** The one-dimensional ^{13}C spectrum.

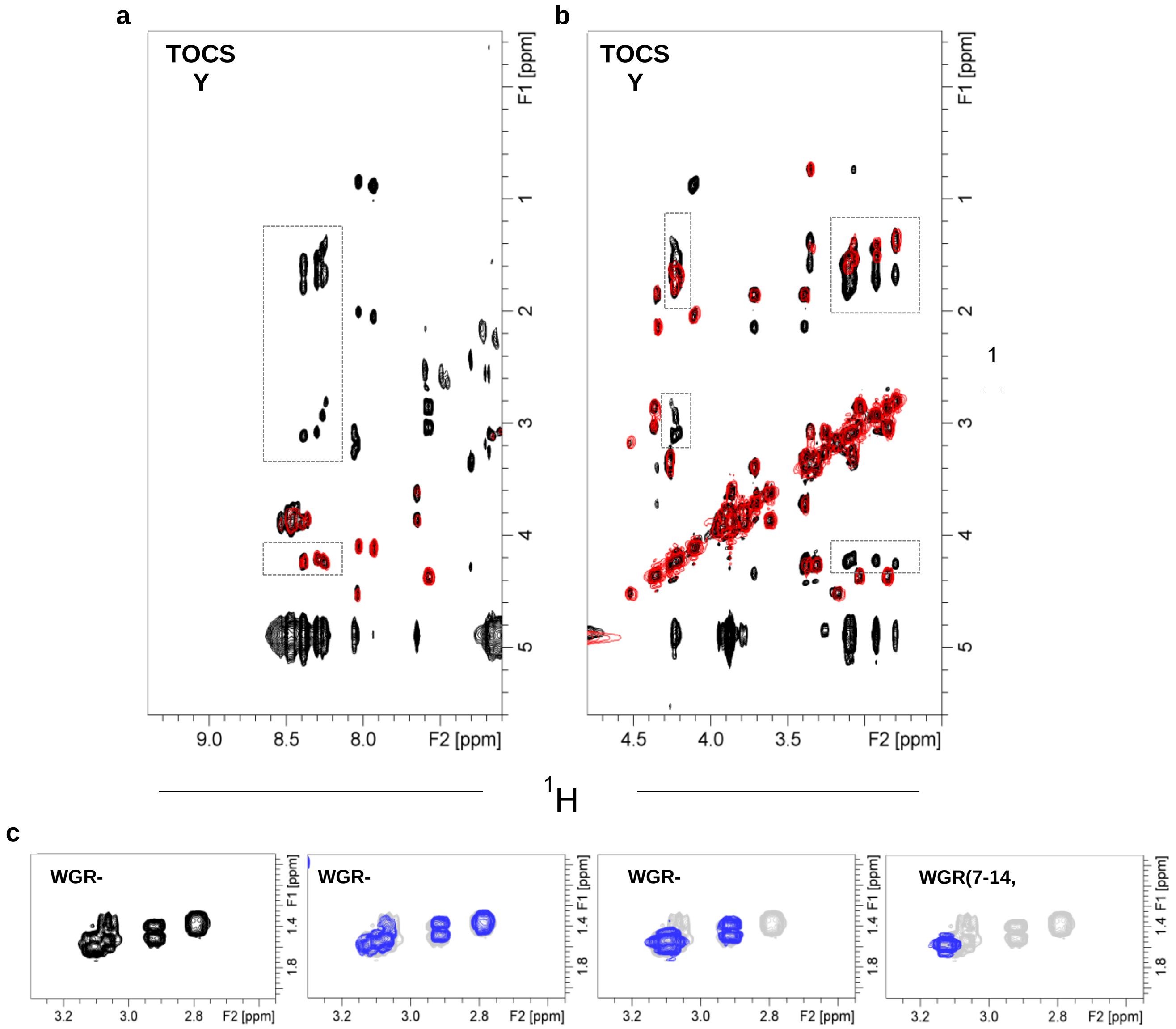

Supplementary Figure 10. Assignment of Arg residues in WGR-1. Shown are spectra used for assignment of the Arg residues of the peptide. **a.** Overlay of homonuclear 2D-TOCSY (black) and COSY (red) spectra in the H^N-H^{ali} region with Arg regions and assignments highlighted. **b.** Overlay of the H^{ali}-H^{ali} region of the same spectra. **c.** Focus on the Arg(H^{γ},H^{δ}) region of the COSY spectrum. Left panel shows this region for WGR-1 (0 M NaCl, pH 6) and successive panels show comparisons of other peptides (blue) to the original peptide (grey). The WGR-2 spectrum demonstrates that upfield shifts are not the result of an interaction with the Y^{14} ring, and the WGR-P10Q spectrum (abolishing the cis/trans conformations) shows that the upfield shift represents a minor conformation (cis). The WGR(7-14, P10Q) spectrum (lacking the R^3/R^5 signals) demonstrates the assignment of R^7.

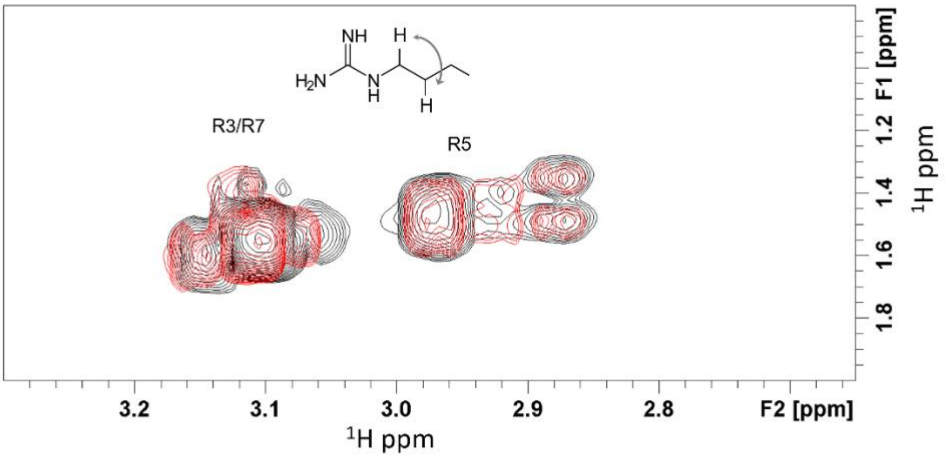

Supplementary Figure 11. NMR determines the molecular mechanism of droplet formation in WGR-3. Region of the 2D-^{1}H, ^{1}H-COSY spectrum showing the correlation between arginine H^{γ}-H^{δ} protons for the WGR-3 peptide at 20 (black, LLPS) and 5 (red, non-LLPS) mM in 50 mM tris buffer pH 10 and 300 K. Changes are observed in the cross-peaks corresponding to Arg^7

Supplementary Table 4.

^{1}H chemical shifts for WGR-1, 20 mM at pH 6, 285 K[a]									
**Residue**	**$^{1}H^{N}$ [ppm]**	**$^{1}H^{\alpha}$ [ppm]**	**$^{1}H^{\beta}$ [ppm]**	**$^{1}H^{\gamma}$ [ppm]**	**$^{1}H^{\delta}$ [ppm]**	**$^{1}H^{\varepsilon}$ [ppm]**	**$^{1}H^{\zeta 2}$ [ppm]**	**$^{1}H^{\zeta 3}$ [ppm]**	**$^{1}H^{\eta 2}$ [ppm]**
W_1	10.25 (10.26)[b,c]	4.27	3.35, 3.41	-	7.30 (7.31)	7.59	7.50	7.16	7.23
R_3/R_5	8.27/8.31	4.23	1.72	1.44	2.93/3.08	-	-	-	-
R_7	8.25	4.26	1.67	1.38	2.80	-	-	-	-
W_9	10.15 (10.24)[b]	4.85 (4.53)	3.09, 3.26 (3.18)	-	7.18 (7.20)	7.61 (7.46)	7.46	7.12	7.21
P_{10}	-	4.35	2.15	(1.34)	3.72, 3.39 (3.35, 3.07)	-	-	-	-
V_{12}	7.94 (8.04)	4.13 (4.11)	2.05 (2.01)	0.89, 0.88 (0.86, 0.83)	-	-	-	-	-
Y_{14}	7.58	4.38	2.86, 3.04	7.02 (7.03)	6.77	-	-	-	-

[a] Glycine residues were not determined due to spectral overlap

[b] Chemical shift for ε1 position

[c] Numbers in brackets represent the chemical shift for the minor isomer

Supplementary Table 5.

^{13}C chemical shifts for WGR-1, 20 mM at pH 6, 285 K[a]

**Residue**	**$^{13}C^{\alpha}$ [ppm]**	**$^{13}C^{\beta}$ [ppm]**	**$^{13}C^{\gamma}$ [ppm]**	**$^{13}C^{\delta 1}$ [ppm]**	**$^{13}C^{\delta 2}$ [ppm]**	**$^{13}C^{\varepsilon 2}$ [ppm]**	**$^{13}C^{\varepsilon 3}$ [ppm]**	**$^{13}C^{\zeta 2}$ [ppm]**	**$^{13}C^{\zeta 3}$ [ppm]**	**$^{13}C^{\eta 2}$ [ppm]**
W_1	56.85	30.88	110.08	128.81	130.07	139.77	121.53	115.49	122.93	125.55
W_9	55.73 (56.74)[b]	29.81 (31.17)	112.08 (111.48)	128.05 (128.21)	130.23 (130.10)	139.51 (139.64)	121.55 (121.47)	115.43	122.93	125.46 (125.60)
P_{10}	64.29 (63.78)	32.62 (33.74)	(24.89)	51.50 (50.62)	-	-	-	-	-	-
V_{12}	63.18 (62.96)	33.54 (33.61)	21.12, 21.82 (20.78, 21.79)	-	-	-	-	-	-	-
Y_{14}	59.91	40.13	132.58	134.03	-	118.73	-	157.69 (157.72)	-	-

[a] Glycines and arginines residues were not determined due to spectral overlap

[b] Numbers in brackets represent the chemical shift for the minor isomer.
