# Supplementary material for: Biomolecular condensates formed by designer minimalistic peptides
Source: Nat Commun. 2023 Jan 26;14:421. doi: 10.1038/s41467-023-36060-8 (PMC9879991; doi:10.1038/s41467-023-36060-8)
Supplement: Supplementary file 3 — Description of Supporting Information [file 41467_2023_36060_MOESM3_ESM.pdf]

Description of Supporting Information

Supplementary Data 1: ^{13}C chemical shifts comparison for WGR-1, 5 & 20 mM, with and without 100 mM NaCl, pH 8, 300 K.
